# Emotional Reactions and Likelihood of Response to Questions Designed for a Mental Health Chatbot Among Adolescents: Experimental Study

**DOI:** 10.2196/24343

**Published:** 2021-03-18

**Authors:** Audrey Mariamo, Caroline Elizabeth Temcheff, Pierre-Majorique Léger, Sylvain Senecal, Marianne Alexandra Lau

**Affiliations:** 1 Department of Educational and Counselling Psychology McGill University Montreal, QC Canada; 2 Department of Information Technologies HEC Montreal Montreal, QC Canada; 3 Department of Marketing HEC Montreal Montreal, QC Canada

**Keywords:** chatbots, conversational agents, mental health, well-being, adolescents, user experience, user preferences

## Abstract

**Background:**

Psychological distress increases across adolescence and has been associated with several important health outcomes with consequences that can extend into adulthood. One type of technological innovation that may serve as a unique intervention for youth experiencing psychological distress is the conversational agent, otherwise known as a chatbot. Further research is needed on the factors that may make mental health chatbots destined for adolescents more appealing and increase the likelihood that adolescents will use them.

**Objective:**

The aim of this study was to assess adolescents’ emotional reactions and likelihood of responding to questions that could be posed by a mental health chatbot. Understanding adolescent preferences and factors that could increase adolescents’ likelihood of responding to chatbot questions could assist in future mental health chatbot design destined for youth.

**Methods:**

We recruited 19 adolescents aged 14 to 17 years to participate in a study with a 2×2×3 within-subjects factorial design. Each participant was sequentially presented with 96 chatbot questions for a duration of 8 seconds per question. Following each presentation, participants were asked to indicate how likely they were to respond to the question, as well as their perceived affective reaction to the question. Demographic data were collected, and an informal debriefing was conducted with each participant.

**Results:**

Participants were an average of 15.3 years old (SD 1.00) and mostly female (11/19, 58%). Logistic regressions showed that the presence of GIFs predicted perceived emotional valence (β=–.40, *P*<.001), such that questions without GIFs were associated with a negative perceived emotional valence. Question type predicted emotional valence, such that yes/no questions (β=–.23, *P*=.03) and open-ended questions (β=–.26, *P*=.01) were associated with a negative perceived emotional valence compared to multiple response choice questions. Question type also predicted the likelihood of response, such that yes/no questions were associated with a lower likelihood of response compared to multiple response choice questions (β=–.24, *P*=.03) and a higher likelihood of response compared to open-ended questions (β=.54, *P*<.001).

**Conclusions:**

The findings of this study add to the rapidly growing field of teen-computer interaction and contribute to our understanding of adolescent user experience in their interactions with a mental health chatbot. The insights gained from this study may be of assistance to developers and designers of mental health chatbots.

## Introduction

### Background

Psychological distress is defined as emotional suffering, characterized by symptoms of depression (ie, sadness, disinterest) and anxiety (ie, tension, agitation) [1]. Longitudinal studies tracking trajectories of psychological distress suggest that they increase across adolescence among both boys and girls [2-4]. Psychological distress has been associated in both meta-analytic and longitudinal studies with important health outcomes such as tobacco use [5,6], drug use [6], and alcohol use [7], with consequences that can extend into adulthood. As such, any interventions aimed at assisting adolescents who may be dealing with psychological distress are of high social importance to reduce their suffering and the consequences associated with distress. One type of technological innovation that may serve as a unique intervention for youth experiencing psychological distress is the conversational agent, otherwise known as a chatbot. Chatbots are “machine conversation systems [that] interact with human users via natural conversational language” [8]. Mental health chatbots are not only increasingly accessible and affordable but may also offer services to individuals who might not seek care due to stigma, elevated cost, or discomfort related to face-to-face therapy [9]. Mental health chatbots have been developed for use among clinical [10,11] and nonclinical [12-14] adult populations. Studies have shown that chatbot users experience improvement in psychological well-being, and tend to find the bots helpful and trustworthy [11,12]. Chatbots geared toward mental health are not only capable of identifying individuals who experience psychological distress but can also help reduce this distress [15]. Furthermore, these agents tend to be rated positively on measures of empathy and alliance [16].

Although chatbots may be deemed more suitable to adolescents, who are more familiar with smartphones [17], most studies on user-chatbot interactions have focused on adults. Among the few studies evaluating mental health chatbots geared toward helping youth, several indicate that these chatbots are effective in the detection and reduction of stress [18], anxiety, and depression [12,13,19]. One study showed that those who consistently interacted with the chatbot seemed to benefit from it [14], suggesting that increasing the likelihood of adherence such as by making these chatbots pleasant to use would be critical in their effectiveness. As such, the focus of this study was to evaluate the factors that increase adolescents’ likelihood of responding to a mental health chatbot and which of its features they perceive more positively.

### Related Research

Researchers have recognized the need for a better understanding of the behaviors and preferences of teens as they increasingly interact with technology [8] and, more specifically, chatbots [20,21]. A review of the literature on human-chatbot interaction highlighted the need for more user-centered research that aims to investigate how and why individuals choose to engage with a particular chatbot [22], as well as how they respond to it.

User experience includes perceptions and responses to the use of a product, as well as any emotions or preferences that occur during its use [23]. Personalizing a chatbot involves customizing its functionality, interface, and content to increase its relevance for an individual or group of individuals [24]. Adherence and engagement are essential to the success of mental health chatbots [25], as they are often associated with better outcomes [14,26]. A chatbot’s characteristics may play an important role in determining whether users will regularly interact with it, as well as whether their interaction will be a pleasant one [27]. The propensity of users to voluntarily share information about themselves is especially crucial for favorable outcomes in their interactions with mental health chatbots. Although the acceptability and effectiveness of these chatbots have been explored [28], little attention has been paid to their characteristics (eg, language, personality) and how they impact user experience.

Two studies that have comprehensively investigated user experience with mental health chatbots described the design and development process of iHelpr, a chatbot that administers self-assessment scales and provides well-being information to adults [29]. The authors not only illustrated the design process but also reviewed the literature on user experience to outline a list of best practices for the design of chatbots in mental health care. Specifically, Cameron and colleagues [29] highlighted adapting the complexity of the chatbot’s language to target users, and varying the content and conversation through the use of GIFs as some of the best practices for mental health chatbots. An evaluation of iHelpr’s usability revealed that participants appreciated its friendly and upbeat personality, and also enjoyed the use of GIFs [29]. A chatbot’s language and the use of graphics such as GIFs are only a few factors to consider when designing such technologies. Emojis, GIFs, and similar media can play a crucial role in determining the framework, sense, and direction of the conversation [25], and may also increase the social attractiveness and credibility of a chatbot [30].

Researchers are beginning to take interest in the effects of graphics on user interactions with mental health chatbots. Fadhil et al [25] showed that users preferred the use of emojis when the chatbot’s questions pertained to their mental health. Duijst [31] reported that participants generally had positive reactions to emojis in a customer service chatbot, suggesting that adding emojis to the chatbot’s dialogue may result in a more pleasant experience. However, some participants felt that combining emojis with a formal tone was strange and inconsistent. Indeed, younger users expressed a preference for a more casual tone, combined with just a few emojis. Adapting a chatbot’s language to its context and users is therefore crucial to improving rapport and user engagement [32]. For instance, chatbots that are expected to be empathic, such as mental health chatbots, may elicit a more positive response from users by communicating in a friendly tone [33,34]. In the context of mental health, where an empathic chatbot would be rated more positively than a less empathic chatbot [35], the use of professional or polite language may be too neutral, possibly leading users to perceive the chatbot as uncaring or indifferent.

### Study Objectives

This study was designed to answer the following question: What are adolescent users’ reactions to questions posed by a mental health chatbot? More specifically, the objective of this study was to evaluate adolescents’ preferences (ie, emotional valence and likelihood of responding) regarding the formulation of questions that might be posed by a mental health chatbot. Preference is indicated by participants’ affective reactions and the likelihood of response to the chatbot’s statements. Given past research suggesting that individuals may prefer emojis and friendly tones in mental health chatbots, the questions presented to participants differed according to their tone (friendly or formal) and the presence of GIFs (present or absent). Questions also differed in type (yes/no, multiple response choice, or open-ended). These factors were chosen based both on past research on mental health chatbots [24,36] as well as the fact that they are easily malleable factors that may improve user experiences. We hypothesized that adolescents would show a preference for questions including GIFs and those with a friendly tone. As the chatbot’s questions also differed according to their type (open-ended or closed), we sought to explore whether adolescents’ preferences would vary in response to question type.

## Methods

### Recruitment

Given that the goal of this study was to assess user preferences for mental health chatbot communication among community adolescents, 19 adolescents aged 14 to 17 years were recruited from the general population via flyers and Facebook advertisements. Participants were informed about the study aims and voluntary participation, and each participant was given compensation of a total value of US $23.74.

### Design and Procedure

**Figure 1:**
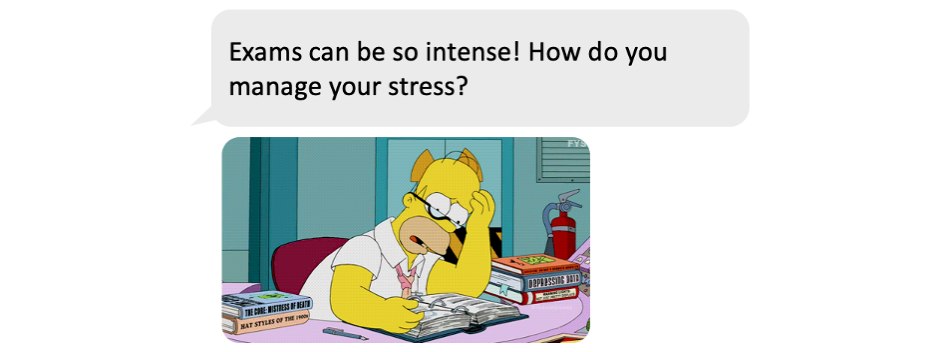
Sample question (friendly tone, open-ended, with GIF).

**Figure 2:**
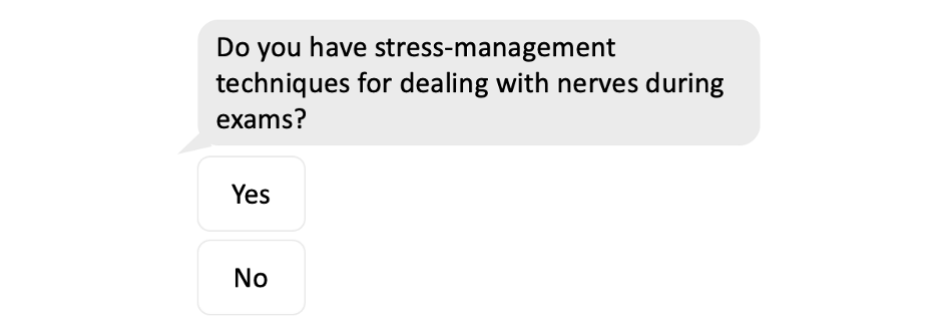
Sample question (professional tone, yes/no, without GIF).

This in-lab study was performed using a 2×2×3 within-subjects factorial design; the factors were presence of GIF (present vs absent), question tone (friendly vs formal), and question type (open-ended vs yes/no vs multiple response choice). Eight main questions were composed (Multimedia Appendix 1), each addressing a different theme centered around general well-being, including mood, stress management, and peer pressure. Each question was modified according to different combinations of each factor, yielding 12 variations for each of the 8 main statements and thus generating a total of 96 questions. The specific topic of each question was maintained across the different variations to control for the effect of theme on users’ reactions. When comparing two levels of one experimental factor (eg, GIF present vs GIF absent), the same question was used for both conditions. The questions and GIFs were developed and pretested by four experts who were experienced in chatbot development. In addition, two adolescents were asked to provide feedback on the proposed questions prior to testing, commenting on readability and understanding of the questions. Sixteen GIFs were evaluated and the final eight (one per main question) were chosen by an expert panel. Sample questions are shown in Figures 1 and 2.

**Figure 3:**
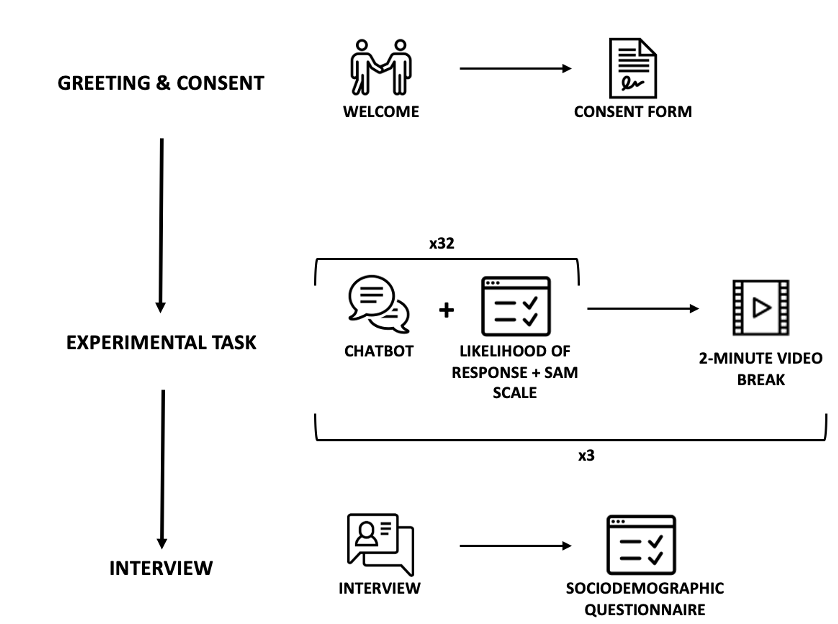
Study procedure. SAM: Self-Assessment Manikin.

Once participants had read and signed the consent form, a research assistant explained the study rationale and gave participants brief verbal instructions. Participants were told to imagine that the questions presented to them were posed by a chatbot that aims to converse with users about their general well-being. Detailed instructions appeared on the computer screen at the start of the study. Participants were encouraged to take their time and to ask questions as needed to ensure they understood the task. All participants were also asked to complete a trial round before beginning the study. Data collection began once participants demonstrated a clear understanding of the task. Each of the 96 questions was presented sequentially on a computer screen for a duration of 8 seconds. Following each presentation, participants were automatically redirected to a short questionnaire presented via Qualtrics (USA) and asked to indicate their likelihood of responding to the question they had just read, as well as their perceived affective reaction to the question. The order of the chatbot questions was randomized for each participant. To prevent participant fatigue, a short 2-minute video was played after each set of 32 questions for a total of two video breaks. At the end of the study, we collected demographic data through another online questionnaire presented via Qualtrics. Informal debriefing was conducted at the end of data collection, and participant feedback was solicited and noted. Data collection lasted between 60 and 90 minutes per participant. An illustration of the study procedure is shown in Figure 3. This study received ethics approval from the Research Ethics Board of HEC Montreal.

### Measures

#### Perceived Emotional Valence

The Self-Assessment Manikin scale is a 9-point nonverbal pictorial assessment tool used to measure the valence associated with one’s affective reactions to stimuli [37]. Valence responses range from sad (1) to happy (9), with lower scores indicating negative valence and higher scores indicating positive valence.

#### Likelihood of Response

Participants’ likelihood of responding to each question was measured with a 5-point Likert scale. Responses ranged from not at all likely (1) to very likely (5). See Multimedia Appendix 2 for the questionnaire used in this study.

### Statistical Analysis

Due to the nonindependent nature of the observations (96 consecutive observations per participant), panel logistic regressions were performed to assess associations between the presence of a GIF, question type, question tone, and each outcome (likelihood of response and perceived valence). Tests were performed while controlling for age, sex, presence of GIF, question type, and tone. The outcome variables were treated as ordinal variables. Regressions were carried out using SAS (version 9.4) and a posthoc power analysis was performed in R. Power analyses revealed that statistical power for the effects of a GIF, question type, and tone on perceived valence was 85%, which is satisfactory, with an odds ratio of 1.35 (β=.30). These analyses also revealed that statistical power for the effects of a GIF, question type, and tone on likelihood of response was 96% with an odds ratio of 1.49 (β=.40).

## Results

### Participant Demographics

Participants were an average of 15.3 years old (SD 1.00) and mostly female (11/19, 58%).

### Perceived Emotional Valence

Question type significantly predicted perceived emotional valence, such that yes/no questions and open-ended questions were associated with a negative perceived emotional valence compared to multiple response choice questions. This suggests that participants had unpleasant affective reactions to yes/no and open-ended questions. Presence of a GIF also predicted perceived emotional valence, such that questions without GIFs were associated with a negative perceived emotional valence, suggesting that questions without GIFs were associated with negative affective reactions. Age group and sex (control variables) did not significantly predict emotional valence, and there was no statistically significant association between tone and perceived emotional valence (Table 1).

**Table 1:** Ordinal logistic regression for factors associated with perceived emotional valence (N=19).

Predictor comparison		β (SE)	*P* value
Presence vs absence (reference) of GIF		–.40 (.09)	<.001
Friendly vs professional (reference) tone		–.15 (.09)	.09
**Question type**			
	Yes/No (reference) vs multiple response choice	–.23 (.10)	.03
	Yes/No (reference) vs open-ended	.03 (.10)	.78
	Multiple response choice (reference) vs open-ended	.26 (.11)	.01

### Likelihood of Response

Question type significantly predicted the likelihood of response, such that yes/no questions were associated with a lower likelihood of response compared to multiple response choice questions and a higher likelihood of response compared to open-ended questions. Furthermore, multiple response choice questions were associated with a significantly higher likelihood of response compared to open-ended questions. Age group was a statistically significant predictor of likelihood of response (β=1.61, *P*=.02), whereas sex was not. Tone and presence of a GIF did not show statistically significant associations with likelihood of response (Table 2).

**Table 2:** Ordinal logistic regression for factors associated with likelihood of response (N=19).

Predictor comparison		β (SE)	*P* value
Presence vs absence (reference) of GIF		–.04 (.09)	.68
Friendly vs professional (reference) tone)		.06 (.09)	.48
**Question type**			
	Yes/No (reference) vs multiple response choice	–.24 (.11)	.03
	Yes/No (reference) vs open-ended	.54 (.11)	<.001
	Multiple response choice (reference) vs open-ended	.78 (.11)	<.001

## Discussion

### Principal Findings

The objective of this study was to investigate adolescents’ preferences regarding question formulation in the context of mental health chatbots. We hypothesized that adolescents would favor questions including GIFs as well as those with a friendly tone. We were also interested in observing whether adolescents preferred certain types of questions over others. Consistent with previous research [36], our results indicate that adolescents’ self-reported affective reactions were significantly more positive in response to questions including GIFs compared to those without GIFs. With respect to question type, participants not only reported more positive affective reactions to multiple response choice questions compared to yes/no and open-ended questions but were also significantly more likely to respond to multiple response questions compared to other question types. The results show that the question features that elicited positive affective reactions did not necessarily lead to a high likelihood of response, and vice versa. For instance, although participants reacted positively to questions with GIFs, the inclusion of GIFs had no statistically significant effect on the likelihood of response.

Participants’ informal verbal feedback provides us with a more nuanced understanding of their experiences and preferences. As reflected in our findings, anecdotal evidence suggests that participants expressed a liking for the inclusion of GIFs in the chatbot’s questions; although they found that GIFs added humor to certain questions, participants did not like all GIFs, and felt that some of these animated images were not relevant to the question with which they were paired. Thus, one possibility is that although participants reacted positively to the GIFs, such images may deter users from responding to certain questions if they are not deemed suitable to the chatbot’s question.

Concerning question type, participants expressed an appreciation for closed questions. Although participants felt that open-ended questions allow them to better express themselves without feeling restricted by predetermined response choices, adolescents found closed questions “easier to respond to.” Interestingly, despite the lack of statistically significant effects for question tone, participants shared positive reflections regarding the friendly tone. In fact, 10 participants specifically mentioned that they enjoyed the use of a friendly tone because it made the chatbot more “relatable” and “human-like,” and 5 participants explicitly stated that they disliked questions with a formal tone. Nevertheless, several participants informally stated that when the chatbot’s tone was overly friendly, it seemed as though the chatbot was “trying too hard.” Furthermore, two participants preferred the formal tone to the friendly one; indeed, these participants felt that the formal tone was more appropriate to the types of questions being posed, whereas the friendly tone gave them the impression that they were not being taken seriously by the chatbot.

### User Experience and Mental Health Chatbots

The findings of this study help us better understand user experience while interacting with a mental health chatbot. The participants’ informal feedback highlights the variability within user preferences and reactions to the features of such chatbots. This variability has been observed in previous research. Yalcin and DiPaola [35] found that user interactions with M-Path, an empathic virtual agent, were not homogeneous. Furthermore, the authors observed that when participants showed more negative emotions, they rated the empathic agent more positively [35], thus illustrating an inconsistency in users’ affective reactions to and ratings of the chatbot. Gaining a better understanding of the function of emotion within user experience is crucial to comprehending user-chatbot interaction, as emotion is closely tied to user acceptance and satisfaction [38] and influences motivation for consumptive behavior [39]. Furthermore, design guidelines for chatbots are generally heterogeneous and largely based on common knowledge rather than on empirical evidence [25]. More often than not, existing chatbots in various domains fail to meet consumer expectations, leading to user frustration and discontinued chatbot use [22,40]. Adolescents are indeed a heterogenous group in many respects and this heterogeneity can be illustrated by the different subcultures that exist among adolescents. Crutzen et al [41] suggest that “subculture-related differences should be taken into account while identifying user needs.” An individual’s personal characteristics also impact their preferences as well as their perceived value of and intention to use a given product. Therefore, to design successful products with specific target users, such as chatbots, developers should be guided by data from the user’s point of view [42].

### Limitations

Several limitations should be considered in the interpretation of these results. The results of this study reflect adolescents’ reactions to potential questions posed by a mental health chatbot used in a voluntary fashion by community adolescents. Thus, these findings may not be generalizable to other chatbots such as customer service agents or mandatory use mental health chatbots. In addition, this study investigated only a few of the many features crucial to chatbot design. Moreover, the results may have been affected by decision fatigue, which can occur when sequential judgments need to be made within a certain time frame. Indeed, asking participants to make multiple ratings or to provide multiple responses in one session could impact subjective usability ratings [43]. However, we do not expect systematic biases in responding, given that the presentation of questions was random and video breaks were inserted into the study protocol. Breaks can be restorative and may “allow a return to original response levels” [44]. Lastly, although the questions and GIFs were pretested by experts, the pretest might have been more thorough if the questions had been pretested using quantitative methods (eg, rated by participants through a survey).

### Conclusions and Future Research

In summary, this study evaluated adolescents’ perceived emotional reactions and likelihood of response to questions posed by a mental health chatbot. These findings add to the rapidly growing field of teen-computer interaction and contribute to our understanding of adolescent user experience in their interactions with a mental health chatbot. A follow-up study should explore which characteristics of GIFs (eg, humor, relevance, size) might play a role in the identified effects, and how user reactions may vary based on different GIFs and based on the different questions posed (ie, the question themes). Future research might also observe users’ back and forth conversations with a prototypical chatbot to investigate design elements that increase user satisfaction and that prolong interaction with the chatbot. The insights gained from this study may be of assistance to developers and designers of mental health chatbots geared toward adolescents. Employing an iterative design process is key to the optimization of mental health chatbots, and evaluating factors that increase user self-disclosure, engagement, and adherence are crucial to the success of these chatbots.

## Appendix

Multimedia Appendix 1 List of questions posed by the chatbot.

Multimedia Appendix 2 Questionnaire presented following each chatbot question.
