# Supplementary material for: Emotional Reactions and Likelihood of Response to Questions Designed for a Mental Health Chatbot Among Adolescents: Experimental Study
Source: JMIR Hum Factors. 2021 Mar 18;8(1):e24343. doi: 10.2196/24343 (PMC8080266; doi:10.2196/24343)
Supplement: Multimedia Appendix 1 [file humanfactors_v8i1e24343_app1.pdf]

Question 1
Theme: How are you?
GIF:

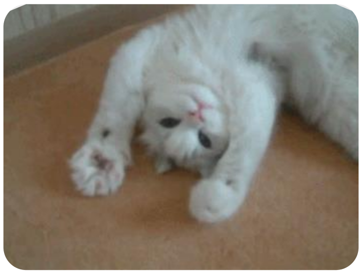

	**Friendly**	**Formal**
**Yes/No**	**Question**<br><br>Hey! You doing good today?<br><br>**Response choices**<br>• Yes<br>• No	**Question**<br><br>Hello! Are you doing well today?<br><br>**Response choices**<br>• Yes<br>• No
**Multiple response choice**	**Question**<br><br>Hey! How you doing today?<br><br>**Response choices**<br>• Super!<br>• Good<br>• Not bad<br>• Meh<br>• Really bad	**Question**<br><br>Hello! How are you doing today?<br><br>**Response choices**<br>• Very good<br>• Good<br>• Average<br>• Not good<br>• Very bad
**Open-ended**	**Question**<br><br>Hey! How you doing today?<br><br>**Response choices**<br>Open-ended	**Question**<br><br>Hello! How are you doing today?<br><br>**Response choices**<br>Open-ended

Question 2
Theme: concentration
GIF:

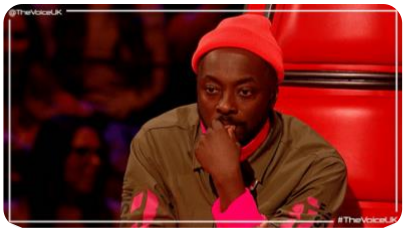

	Friendly	Formal
**Yes/No**	**Question**<br><br>Do you ever find yourself daydreaming?<br><br>**Response choices**<br>• Yes<br>• No	**Question**<br><br>Do you ever have trouble concentrating?<br><br>**Response choices**<br>• Yes<br>• No
**Multiple response choice**	**Question**<br>Do you ever find yourself daydreaming?<br><br>**Response choices**<br>• Never<br>• Sometimes<br>• Often<br>• Huh? What did you say?	**Question**<br>Do you ever have trouble concentrating?<br><br>**Response choices**<br>• Never<br>• Occasionally<br>• Frequently<br>• Always
**Open-ended**	**Question**<br>It sucks, but sometimes people have a hard time concentrating. When does that happen to you?<br><br>**Response choices**<br>Open-ended	**Question**<br>Sometimes a person may have difficulty concentrating. In what situations does this happen to you?<br><br>**Response choices**<br>Open-ended

Question 3
Theme: smoking cigarettes
GIF:

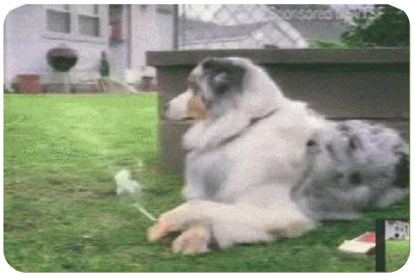

	Friendly	Formal
**Yes/No**	**Question**<br>Be real! Have you ever wanted to smoke a cigarette?<br><br>**Response choices**<br>• Yes<br>• No	**Question**<br>Have you ever felt the urge to smoke a cigarette?<br><br>**Response choices**<br>• Yes<br>• No
**Multiple response choice**	**Question**<br>I'm curious: what makes you want to smoke a cigarette the most?	**Question**<br>What kinds of situations make you smoke the most?

	**Response choices**<br>• Being with friends who smoke<br>• Being at a party<br>• I never want to smoke	**Response choices**<br>• Being around friends who smoke<br>• Social events<br>• I never have the urge to smoke
**Open-ended**	**Question**<br>I'm curious: what makes you want to smoke a cigarette the most?<br><br>**Response choices**<br>Open-ended	**Question**<br>What kinds of situations make you smoke the most?<br><br>**Response choices**<br>Open-ended

Question 4
Theme: parents
GIF:

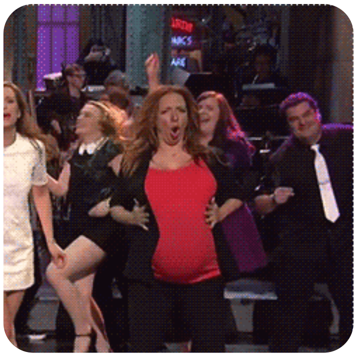

	Friendly	Formal
**Yes/No**	**Question**<br>Do you get along well with your parents?<br><br>**Response choices**<br>• Yes<br>• No	**Question**<br>Do you have a good relationship with your parents?<br><br>**Response choices**<br>• Yes<br>• No
**Multiple response choice**	**Question**<br>Your parents, do you get along well with them?<br><br>**Response choices**<br>• Really well!<br>• Not so bad<br>• Could be better<br>• I dunno, not so good	**Question**<br>Do you have a good relationship with your parents?<br><br>**Response choices**<br>• Very good<br>• Good<br>• Average<br>• Bad
**Open-ended**	**Question**<br>Say, how do you get along with your parents?<br><br>**Response choices**<br>Open-ended	**Question**<br>How would you describe your relationship with your parents?<br><br>**Response choices**<br>Open-ended

Question 5
Theme: the future
GIF:

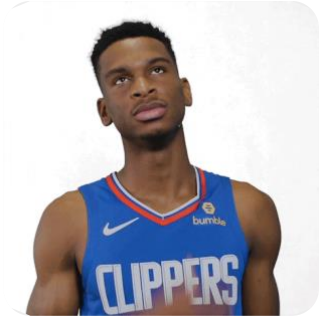

	Friendly	Formal
**Yes/No**	**Question**<br>Say, do you often think about the future?<br><br>**Response choices**<br>• Yes<br>• No	**Question**<br>Do you ever have thoughts about the future?<br><br>**Response choices**<br>• Yes<br>• No
**Multiple response choice**	**Question**<br>Say, do you often think about the future?<br><br>**Response choices**<br>• Never<br>• It happens<br>• Pretty often<br>• Ugh! All the time!	**Question**<br>Do you ever have thoughts about the future?<br><br>**Response choices**<br>• Never<br>• Rarely<br>• Frequently<br>• Always
**Open-ended**	**Question**<br>What do you think about your future?<br><br>**Response choices**<br>Open-ended	**Question**<br>How do you feel when thinking about your future?<br><br>**Response choices**<br>Open-ended

Question 6
Theme: peer pressure
GIF:

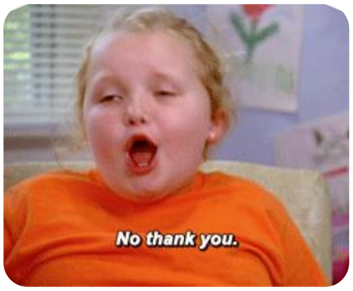

	Friendly	Formal
**Yes/No**	**Question**<br>Have you ever agreed to do something because your friends made you feel bad for saying no?<br><br>**Response choices**<br>• Yes<br>• No	**Question**<br>Have you ever felt pressured into doing something out of fear of your friends' reactions?<br><br>**Response choices**<br>• Yes<br>• No
**Multiple response choice**	**Question**<br>Have you ever agreed to do something because your friends made you feel bad for saying no?<br><br>**Response choices**<br>• Never<br>• It happens<br>• Pretty often<br>• All the time	**Question**<br>Have you ever felt pressured into doing something out of fear of your friends' reactions?<br><br>**Response choices**<br>• Never<br>• Rarely<br>• Frequently<br>• Always
**Open-ended**	**Question**<br>What do you do when your friends make you feel bad for saying no to an activity?<br><br>**Response choices**<br>Open-ended	**Question**<br>How do you react when your friends insist that you do an activity that you don't want to do?<br><br>**Response choices**<br>Open-ended

Question 7
Theme: sleep
GIF:

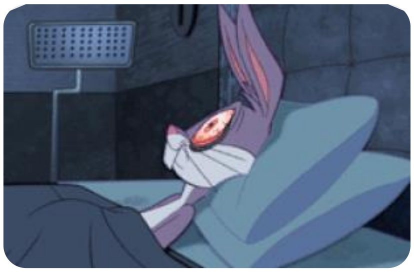

	Friendly	Formal
**Yes/No**	**Question**<br>Say, do you ever have trouble sleeping because you're feeling stressed?<br><br>**Response choices**<br>• Yes<br>• No	**Question**<br>Do you ever lose sleep as a result of worrying?<br><br>**Response choices**<br>• Yes<br>• No
**Multiple response choice**	**Question**<br>Say, do you ever have trouble sleeping because you're feeling stressed?<br><br>**Response choices**<br>• All the time<br>• It happens<br>• Pretty often<br>• Zzzz… huh?	**Question**<br>Do you ever lose sleep as a result of worrying?<br><br>**Response choices**<br>• Never<br>• Rarely<br>• Frequently<br>• Very frequently
**Open-ended**	**Question**<br>What kinds of things keep you up at night?<br><br>**Response choices**<br>Open-ended	**Question**<br>Under what circumstances do you lose sleep?<br><br>**Response choices**<br>Open-ended

Question 8
Theme: dealing with stress/anxiety during exams
GIF:

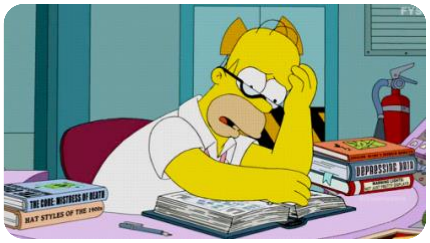

	Friendly	Formal
**Yes/No**	**Question**<br>Exams can be so intense! Do you have tricks to deal with your stress?<br><br>**Response choices**<br>• Yes<br>• No	**Question**<br>Do you have stress-management techniques for dealing with nerves during exams?<br><br>**Response choices**<br>• Yes<br>• No
**Multiple response choice**	**Question**<br>Exams can be so intense! How do you deal with your stress?<br><br>**Response choices**<br>• I play sports<br>• I meditate<br>• I hang out with friends<br>• I study hard	**Question**<br>What stress-management techniques do you use to deal with nerves during exams?<br><br>**Response choices**<br>• Playing sports<br>• Meditation<br>• Seeing friends<br>• Studying as much as possible
**Open-ended**	**Question**<br>Exams can be so intense! How do you manage your stress?<br><br>**Response choices**<br>Open-ended	**Question**<br>What stress-management techniques do you use to deal with nerves during exams?<br><br>**Response choices**<br>Open-ended
