# Supplementary figures and images for: Emotional Reactions and Likelihood of Response to Questions Designed for a Mental Health Chatbot Among Adolescents: Experimental Study
Source: JMIR Hum Factors. 2021 Mar 18;8(1):e24343. doi: 10.2196/24343 (PMC8080266; doi:10.2196/24343)

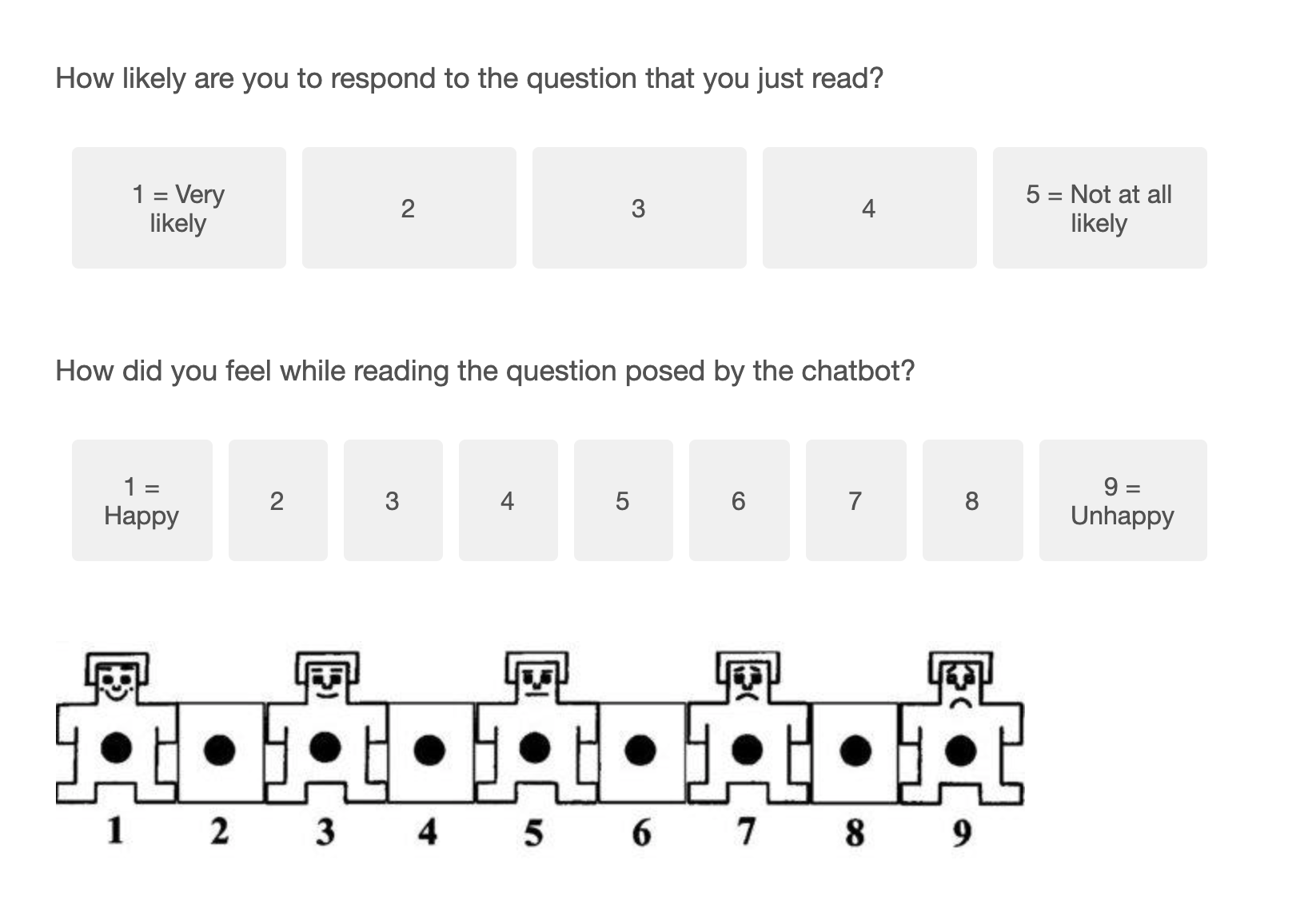

Supplement: Multimedia Appendix 2 [file humanfactors_v8i1e24343_app2.png]
